# “Maintaining symbiosis in conflict”: the quality of life of disabled elderly individuals in Chinese elderly care institutions – a grounded theory study

**DOI:** 10.1080/17482631.2024.2397845

**Published:** 2024-09-05

**Authors:** Ying Zuo, Guang Yang

**Affiliations:** School of International and Public Affairs, Shanghai Jiao Tong University, Shanghai, China

**Keywords:** Ageing, disabled elderly individuals, elderly care institution, quality of life, grounded theory study

## Abstract

**Background and Objectives:**

Exploring the quality of life of disabled elderly individuals in eldercare facilities holds significant importance in the improvement of service quality, the allocation of eldercare resources, and the enhancement of the well-being of the elderly. This study, grounded in the subjective perspective of disabled elderly individuals, aims to investigate their quality of life within eldercare institutions.

**Research Design and Methods:**

A grounded theory approach was employed, involving semi-structured interviews with 35 participants.

**Results:**

Data analysis revealed that the quality of life of disabled elderly individuals in Chinese elderly care institutions is characterized by “maintaining symbiosis in conflict” and encompasses four dimensions: complex adaptation process, complexities in social interactions, physical pain and the lonely soul.

**Discussion and Implications:**

Spending late years in elderly care institutions poses a trial and challenge for disabled elderly individuals, especially within a cultural environment that traditionally revolves around the “family” unit. In these institutions, disabled elderly individuals not only endure physical pain but also grapple with feelings of loneliness. They maintain the facade of family dignity by concealing true emotions, ensuring the harmonious and stable operation of the elderly care institution.

Enhancing the quality of life for disabled elderly individuals requires not only an improvement in the service capabilities of elderly care institution staff but also collaborative efforts from policymakers and family members.

## 1. Introduction

With advancements in medical technology and improvements in living standards, human life expectancy has steadily increased, leading to a significant global ageing phenomenon. According to United Nations data, the global population aged 65 and above is projected to reach 1.6 billion by 2050, constituting 16% of the total population (He et al., 2016). China, as the most populous country in the world, is also experiencing rapid ageing. According to the 7th National Population Census of China, the population aged 65 and above constitute 190 million people, making up 13.50% of the total population (National Bureau of Statistics, 2021). The 2018 Fourth National Survey of the Living Conditions of the Elderly in Urban and Rural Areas in China reveals that the average life expectancy is 77 years, with a healthy life expectancy of only 68.7 years, indicating a significant gap (Yang & Meng, 2020). The number of elderly individuals with disabilities is on the rise, making caregiving for disabled seniors a prominent concern within the field of elderly care. By theories of elderly migration, seniors who are severely disabled or incapable of self-care often transition into institutionalized environments (Bradley, 2011; Rijnaard et al., 2016).

To address the care needs of elderly individuals with disabilities, both public and private elderly care institutions in China have rapidly developed with government support. The number of institutions and available beds has significantly increased. By the end of 2020, there were 38,158 registered elderly care institutions in China, with a total of 4.882 million beds and 2.224 million residents. Among these residents, 491,000 were fully disabled, and 607,000 were partially disabled. The ratio of public to private institutions is approximately 1:1. From 2010 to 2020, the number of elderly care institutions accommodating semi-disabled and disabled elderly individuals grew from 518,000 to 1.098 million, with the proportion rising from 21.35% to 49.38%, reflecting the gradual improvement in care capacity. In terms of staffing, the caregiver-to-resident ratio in Chinese elderly care institutions improved from one caregiver for every six residents in 2016 to one caregiver for every four residents in 2020. According to data from the Ministry of Civil Affairs, by the end of 2020, there were 615,000 employees in elderly care institutions nationwide, including approximately 322,000 caregivers (China Civil Affairs Statistical Yearbook, 2021). However, there remains a significant shortage of skilled elderly care personnel. To better support and guide the development of elderly care institutions, China has established a standard system for elderly care services, encompassing aspects such as facility modifications for elderly accessibility, care services, and staffing requirements. Both public and private institutions must adhere to these national standards and regulations and are subject to strict oversight by regulatory authorities. Additionally, in the “14th Five-Year Plan,” the Chinese government has set mandatory targets for elderly care institutions, requiring that at least 45% of their beds be dedicated to care services (Outline of the Fourteenth Five-Year Plan for the National Economic and Social Development of the People’s Republic of China and the Vision, 2021). This demonstrates the government’s strong commitment to improving the care and quality of life for elderly individuals with disabilities, mobilizing diverse resources to address current shortcomings in the elderly care market.

Quality of Life (QoL) has emerged as a central benchmark in clinical practice and research within eldercare institutions (Kane et al., 2005) garnering extensive attention from scholars. In recent years, researchers employ various QoL assessment tools such as the Short Form-36 (SF-36) (Sima et al., 2021) WHOQOL-BREF (Lucas-Carrasco et al., 2011) the Quality of Life Questionnaire (EQ-5D) (Toh et al., 2021) and the World Health Organization Quality of Life Instrument-Older Adults Module (WHOQOL-OLD) to evaluate the quality of life of elderly individuals residing in elderly care institutions (Sandgren et al., 2021). However, the scales used in such studies are generic and health-related, primarily focusing on quantifiable indicators that pertain to health (Hansen, 2020). While these aspects are highly relevant to the QoL of elderly individuals in care facilities (Fleming et al., 2016; Shogren et al., 2017) they only capture one dimension of subjective importance to the elderly residents (Mondaca et al., 2019) thus underestimating the multidimensionality of QoL (Pequeno et al., 2020). Scholars have increasingly recognized the significance of the subjective experiences of elderly residents in care facilities (Bowling et al., 2003; Brandburg et al., 2013). Many scholars have started to determine the dimensions of QoL for elderly residents in care facilities based on their subjective feelings (O’Neill et al., 2022; Shogren et al., 2017; Sion et al., 2020). Furthermore, additional research suggests that when transitioning from their homes to care facilities, elderly individuals often experience a sense of “You’re at their mercy”, which significantly impacts QoL (O’neill et al., 2020). Nonetheless, elderly residents in care facilities continue to actively “seek and maintain connections” connections (Gautam et al., 2022) not only with family members (Heller et al., 2015) but also with the staff and fellow residents in the facility (Roberts & Bowers, 2015). Through these interactions, they develop a new sense of identity and meaning in life (Cater et al., 2022). These investigations affirm the intricate and subjective nature of QoL, emphasizing the need to place individuals at the forefront of quality of life research (Vanleerberghe et al., 2017).

However, existing research has largely overlooked the exploration of the QoL of disabled elderly individuals in nursing homes. In addition to enduring the common difficulties faced by other elderly individuals, they also bear significant psychological burdens and moral pressures (Chou, 2010; Zhan et al., 2008). Therefore, our research aims to explore and identify the key dimensions of quality of life for disabled elderly individuals in Chinese elderly care institutions from an individual perspective through qualitative interviews. We strive to identify key factors that significantly impact their quality of life, enhancing the understanding of the living conditions of disabled elderly residents in these institutions.

## 2. Methods

### 2.1. Research design

The QoL for elderly individuals with disabilities residing in elderly care institutions offers a unique research perspective defined by intricate and dynamic processes. This perspective is best examined through qualitative research. Grounded theory serves as an apt approach for qualitative research, requiring researchers to extract experiential abstractions directly from empirical observations or raw data. These abstractions subsequently coalesce into a systematic theoretical framework (Strauss & Corbin, 1998; Wuetherick, 2010). The procedural grounded theory employs a three-stage coding process: open coding, axial coding, and selective coding. This approach offers greater structure, procedural rigour, and standardization, making it widely used. This study follows the research framework of procedural grounded theory (Corbin & Strauss, 1990). In this study, we employ procedural grounded theory and adhere rigorously to the prescribed coding standards inherent in grounded theory methodology.

### 2.2. Participants

The elderly care institutions in China primarily comprise publicly funded and privately operated types. To ensure the scientific validity of our research, we selected one publicly funded and one privately operated institution in H City. LY is a universally accessible publicly funded elderly service institution established with government investment. LK is a privately operated elderly care institution integration.

According to international standards, if an individual fails to meet the criteria in any of six indicators, including eating, dressing, using the toilet, getting in and out of bed, indoor mobility, and bathing, they can be classified as “disabled.” Specifically, inability to perform one to two of these tasks is categorized as “mild disability,” three to four as “moderate disability,” and five to six as “severe disability.” This study adheres to these widely accepted standards in assessing disabled older adults (Burke, 1996). Meanwhile, in China, the government has officially issued the national standard “Specification for Assessing the Abilities of Elderly Individuals” (GB/T42195-2022). All disabled elderly individuals undergo a standardized assessment upon admission to elderly care institutions. Researchers use this standard as a reference when selecting study subjects. The basic inclusion criteria for disabled elderly individuals are as follows: (1) Residents aged 60 years or older; (2) Residing for at least six months; (3) Conformance to international common standards for disabled elderly individuals, excluding severe cognitive impairment; (4) Voluntary willingness to engage in interviews. The study involved a collective sampling of 35 elderly individuals with disabilities. Specifically, 18 elderly individuals with disabilities were selected from the LY Elderly Care Institution, and interviewees were assigned unique identifiers from LY01 to LY18. Additionally, 17 elderly individuals with disabilities were selected from the LK Elderly Care Institution, all interviewees were assigned distinct identifiers from LK01 to LK17.Essential demographic information is provided in Table I for reference.

**Table I.** Basic socio-demographic data.

Category	Characteristics	sample size	proportion
Gender	Male	17	48.57%
	Female	18	51.43%
Age	60-70 years	3	8.57%
	70-80 years	6	17.14%
	80-90 years	18	51.43%
	>90 years	8	22.86%
Length of residence in home	6 months–1 years	7	20.00%
	1 years—3 years	24	68.57%
	>3 years	4	11.43%
Residential Situation	Single occupancy room	2	5.71%
	Double occupancy room	19	54.29%
	multi-occupancy room	14	40.00%
Degree of disability	Mild disability	11	31.43%
	Moderate disability	12	34.29%
	Severe disability	11	31.43%
Marital Status	Widowed	26	74.29%
	Married	9	25.71%

### 2.3. Ethics approval

The study protocol was approved by the ethics review committee of Shanghai University of Medicine & Health Sciences, China based on the assessment of design and process of the study and was conducted in accordance with the principles of the Declaration of Helsinki. Before starting the research study, the respondents were informed about the anonymity of this study with relevant details. The questionnaire-related information is as follows: “In order to ensure the authenticity and confidentiality of the research, this research is anonymous, and the content of the survey is only used for relevant research, and the content of your answers will be kept confidential in accordance with the requirements of the Statistical Law of the People’s Republic of China”.

The study participants were provided in detail the purpose of the study, analysis of the contents of the study, interview method, and recording contents. Data were collected after obtaining written consent. The collected data were not used for any purpose other than the research, the consent form was kept in a locked cabinet, and the transcribed data were stored in the researcher’s computer with a password.

### 2.4. Data collection

In our study, we gathered primary data by conducting semi-structured, in-depth interviews with a cohort of 35 elderly residents hailing from diverse elderly care institutions. These discussions occurred within the privacy of the participant’s room. We opted to conduct interviews with disabled elderly individuals residing in double or multiple occupancy rooms when their roommates were absent. The duration of each interview ranged from approximately 30 to 60 minutes. Our interview protocol was designed as a semi-structured format, with a primary focus on three key themes. Concurrently, during the research process, differential probing was conducted based on the varied responses of disabled elderly individuals.

Firstly, how do you assess the QoL experienced in your current residential setting?

Secondly, what factors exert influence on the dimensions of your life quality in this context?

Thirdly, in what specific facets do you aspire to witness improvements for an enhanced QoL?

### 2.5. Data analysis

Data analysis commences immediately following the initial interview and is carried out following each subsequent interview, preceding the subsequent one. Concurrently, by the research framework rooted in procedural grounded theory, to progressively refine and synthesize the original data through three distinct stages of coding: Open Coding, Axial Coding, and Selective Coding. We continuously conduct comparisons to guide the data analysis, exploring the relationships among data points and theories while continually scrutinizing data and theories. This rigorous process is aimed at elucidating, predicting, and exploring the logical connections between various conceptual categories within the original data (Hallberg, 2006) ultimately culminating in the emergence of novel theoretical constructs once theoretical saturation is attained (Glaser & Strauss, 1965; Suddaby, 2006). In this study, when data analysis no longer yielded new categories, the researchers continued interviewing three disabled elderly individuals to verify if data saturation had been achieved (Francis et al., 2010). The coding outcomes revealed no emergence of new categories. Consequently, the study successfully passed the test for theoretical saturation.

### 2.6. Research validity and reliability

Credibility in qualitative research pertains to the rigour of the study design, the trustworthiness of the researchers, the credibility of the research outcomes, and the applicability of the research methods (Johnson & Parry, 2022). To ensure credibility in this study, several measures were implemented. Firstly, the study design was meticulously crafted. The researchers engaged in this project for an extended period and conducted prolonged observations of the participants, based on semi-structured interviews. This approach provided in-depth insights into the actual living experiences of the participants in elderly care institutions. Secondly, to maintain the credibility of the researchers, two experienced professionals well-versed in the theoretical framework employed herein were tasked with independently coding the interview data. Additionally, Expert opinions were sought when necessary, from professors with extensive research and practical experience in the field of elderly care. Thirdly, the credibility of the research outcomes was established as the study reached theoretical saturation. The emerging concepts, categories, and their interrelationships were consistently validated during interviews, underscoring the high authenticity and value of recurrent concepts and categories (O’Neill et al., 2022). Lastly, the applicability of the research methods was maintained by leveraging the richness of the data obtained through in-depth interviews (Engward, 2013).

## 3. Results

**Figure 1.**
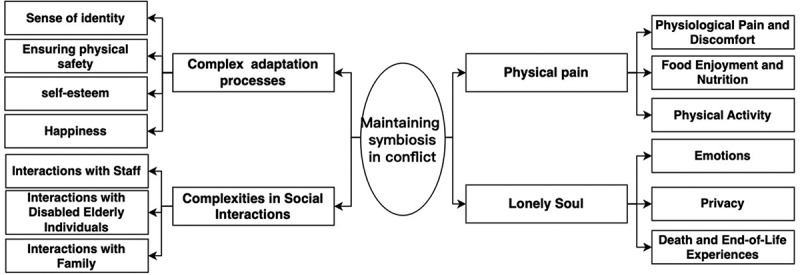
Dimensions of quality of life reported by participants.

Through the analysis of interview materials, four core dimensions were identified: Complex Adaptation Processes, Complexities in Social Interactions, Physical Pain, and Lonely Soul. Each dimension comprises various sub-dimensions (see Figure 1). In this section, we will discuss each dimension in detail.

### 3.1. Complex adaptation processes: transition from home to elderly care institutions

#### 3.1.1. Sense of identity

When most elderly individuals with disabilities first enter institutions, they tend to isolate themselves and are reluctant to interact with other residents. However, as they become more familiar with their surroundings, they seek a sense of identity through two main approaches, which helps them integrate into the new environment more quickly. On one hand, disabled elderly individuals, affected by various illnesses, are particularly concerned with the basic facilities and medical provisions of elderly care institutions. *“The place is well-maintained, and it makes me feel comfortable” (LY11)*. Simultaneously, the physical environment is thoughtfully adapted to accommodate the functional abilities and mobility characteristics of the elderly through age-appropriate modifications. Furthermore, the emphasis on whether the residential care facility is equipped with medical professionals, such as doctors and nurses, is a significant concern for disabled elderly individuals. *“There are doctors and nurses here, and for minor ailments, you can receive medical attention here. I highly appreciate this” (LK12)*. Disabled elderly individuals construct a positive “image” of nursing homes through their evaluation of the physical environment. Additionally, by comparing themselves to others, they develop a sense of relative well-being. *“When I see the condition of many elderly people here, I feel that my situation is much better” (LY24)*. This process of comparison enables some disabled elderly individuals to shed the negative identity of being a “troublemaker” and gradually reconcile with their newly constructed self-identity.

#### 3.1.2. Ensuring physical safety

Most disabled elderly individuals end up relocating to elderly care institutions due to a lack of sufficient care at home. *“I could not take care of myself at home, I came to this place. At first, I was sad, but I told myself that it’s better than being alone” (LY18)*. Moving into elderly care institutions is perceived by disabled elderly people as a gateway to a safer living environment where basic care is assured. *“Before moving here, I lived with my daughter, but she had to work during the day, and I was often unable to get up by myself if I fell. There’s someone to take care of me all day” (LY03)*. Nevertheless, some disabled elderly individuals point out that they often experience long wait times when they need assistance to use the restroom during the night, increasing their risk of falls. Additionally, their incontinence issues during the night may go unnoticed, affecting their sleep quality and causing discomfort.

#### 3.1.3. Self-esteem

Disabled elderly individuals desire to maintain and continue their habits from when they were living at home. Some of the regulations in elderly care institutions, due to lack of necessary flexibility, to a certain extent, deprive disabled elderly individuals of their personal choices. An elderly individual expressed dissatisfaction with a perceived lack of control over meal schedules, stating, *“They arrange meals at specific times, and if you miss the designated time, there is no food available” (LK14)*. Many disabled elderly individuals do not appreciate the disruption of their pre-existing routines but are compelled to adhere to the facility’s schedule. Meanwhile, disabled elderly individuals desire to retain as much control over their lives as possible within the confines of care institutions. They seek to maintain a degree of self-sufficiency to feel valuable, but this need is often not respected. *“I can move my hands, dress myself, and do some tasks on my own, which is very meaningful to me. However, they think I’m too slow, and I have to follow their arrangements” (LY09)*. This lack of autonomy causes them to lose their sense of control over their lives. Additionally, as their circumstances change, disabled elderly individuals may develop new habits and preferences. However, elderly care institutions continue to provide fixed services based on the information provided by their family members at the time of admission. *“They listen to my child, and all decisions about me are made through direct communication with them. They don’t ask for my opinion” (LY06)*. This results in a shift from autonomous decision-making to pseudo-autonomous decision-making. The opinions of disabled elderly individuals are neither solicited nor integrated into their care plans. Consequently, these individuals must live strictly according to the institution’s regulations, leading to feelings of disrespect and disregard.

#### 3.1.4. Happiness

Elderly care institutions celebrate national holidays such as the Spring Festival, and Mid-Autumn Festival, and other meaningful occasions like Mother’s Day and Father’s Day through various activities. The cultural values of endurance, acceptance, and gratitude in Chinese society significantly influence the attitudes of disabled elderly residents in care institutions. Nearly all of these residents express profound gratitude for the care they receive and feel fortunate that staff members organize activities for them. One elderly resident vividly expressed her experience on Mother’s Day: *“On Mother’s Day, the staff came to take photos of us, and they gave us flowers. I’m very grateful. When they call me ‘grandma,’ it makes me very happy” (LK07)*. Collective activities have transformed the otherwise idle lives of elderly individuals with disabilities, fostering opportunities for communication between them and staff members while enhancing their sense of pleasure. In addition, personnel in elderly care institutions can enhance the sense of value for disabled elderly individuals by implementing targeted personal activities. *“Our director sometimes pushes me for a stroll outside, drives me to the supermarket, and I can buy things I like, which makes me very happy” (LY15)*. These meaningful activities foster a family-like atmosphere and substantially enhance the previously inactive lives of elderly individuals with disabilities. They offer increased opportunities for interaction between staff and residents, thereby improving mutual understanding and trust. As a result, life in care institutions becomes more manageable and enjoyable for disabled elderly individuals.

### 3.2. Complexities in social interactions

#### 3.2.1. Interactions with staff in elderly care institution

In the context of elderly care institutions, these institutional caregivers are predominantly perceived as providers of nursing services, rather than individuals fostering meaningful relationships with the residents. Disabled elderly individuals tend to establish shallow, informal relationships with staff, striving to cooperate seamlessly with them to facilitate effective collaboration. The elderly residents must avoid being seen as burdensome by the staff. *“Everyone is taken care of here, they try their best to meet our needs, but I also don’t want to make too many demands” (LK16)*. When elderly residents seek assistance from staff members, they must also interpret the staff members’ facial expressions and body language to assess emotional state. Many disabled elderly individuals express dissatisfaction with the staff members’ indifference and unfriendliness. Nevertheless, these elderly residents tend to “conceal their discontent” and remain silent, fearing potential reprisals. *“Whether I speak positively or negatively, once you leave, they may retaliate. What can we do? We are elderly, and at this age, we are at the mercy of others.” (LK09).*

#### 3.2.2. Interactions with disabled elderly individuals

Building relationships among disabled elderly individuals in elderly care institutions is fraught with difficulties. The majority of these elderly individuals lack the physical capacity to establish connections, *“It’s challenging to communicate with them. They’re all either bedridden, physical disability, or mentally disoriented” (LK01)*. Furthermore, a more alarming issue surfaces in the form of “daily bullying” among disabled elderly residents. *“There are several mentally unstable ones,sometimes they push others, especially those with severe illnesses” (Interview Data: LK17)*. Compounding this issue is the tacit acceptance of bullying within elderly care institutions. Some adult children of partially disabled elderly individuals offer “additional” gifts to caregivers and staff, hoping for increased tolerance and care for their parents. This “gift-giving” phenomenon, influenced by Chinese culture, is prevalent in both public and private elderly care institutions. Conversely, when these disabled elderly individuals display “bullying” behaviour towards other elderly individuals whose children have not provided gifts, caregivers and staff often choose to remain passive rather than intervening. Management personnel and staff exhibit inadequate concern for instances of “bullying” *“They can’t say anything because many elderly residents’ children give them additional “gifts,” so they take extra care of these residents” (LY12)*. To minimize conflicts, a prevalent strategy emerges—the collective choice to “ignore.” Maintaining silence becomes the chosen resolution strategy for unpleasant conflicts among disabled elderly individuals. This “gift-giving” phenomenon, influenced by Chinese culture, is prevalent in both public and private elderly care institutions. The prevalence of adverse emotions renders the lives of disabled elderly individuals akin to “walking on thin ice.”

#### 3.2.3. Interactions with family

The presence of family holds paramount significance for the majority of disabled elderly individuals. This companionship assures them that they have not been forgotten. Disabled elderly individuals often refrain from disclosing their unfortunate experiences in these institutions to their offspring. *“I usually tell them everything is fine. I don’t want them to take time off work to visit me; I don’t want them to go through too much trouble on my account” (LK06)*. However, the genuine sentiments harboured within disabled elderly individuals reveal a desire for their offspring to visit them more frequently.*“When I see them celebrating holidays, with their children all present, and no one comes to visit me, it makes me very sad” (LY14)*. Many family members, unable to visit regularly, resort to video calls with their elderly relatives. *“My daughter calls me on video, the phone screen is too small, and I can’t hear well; it’s not very effective” (LK11)*. Emotional connections conveyed through remote video transmission fall short of replacing actual visits, as disabled elderly individuals yearn for the physical presence of their children.

### 3.3. Physical pain

#### 3.3.1. Physiological pain and discomfort

The fatigue experienced by frail elderly individuals in elderly care institutions is intricate, multifaceted, and overwhelming, often accompanied by feelings of depression and despondency. *“I feel tired every day, I even hesitate to use the restroom. Moving from the bed to a wheelchair or the bathroom requires significant time and energy” (LY04)*. The majority of elderly individuals experiencing functional impairments no longer aspire for disease cure; instead, their desire is focused on alleviating pain.However, this issue has not yet received adequate attention and proper handling within elderly care institutions, leading to prolonged suffering. *“I’m in pain. I’ve visited multiple hospitals, and the doctors say there’s no cure” (LK15)*. Many elderly individuals with disabilities unable to perceive external sounds effectively, *“My ears are no good; communication is challenging.” (LY08)*. The loss of sensory and functional abilities deprives these frail elderly individuals of opportunities to communicate with the outside world, exacerbating their sense of fatigue.

#### 3.3.2. Food enjoyment and nutrition

The majority of disabled elderly individuals require food and nutrition to meet high standards. Currently, the communal meals offered by elderly care institutions fail to adequately fulfil the nutritional needs of disabled elderly individuals. *“I have no choice but to eat it. Like last night, they made that noodle soup, there were only noodles in the water, no vegetables, just a bit of saltiness. So, I didn’t eat last night” (LY01)*. Many elderly individuals with disabilities require special diets. For instance, those who have lost their teeth and struggle with chewing prefer soft and mushy food. However, the challenge lies in making such food both palatable and satisfying, *“I don’t have teeth, I can’t bite. They turn the meat into porridge to feed me, and it’s so unappetizing that I can’t swallow it” (LY15)*. Furthermore, some severely disabled elderly individuals require assistance from staff during meal times. A staff member may have to feed multiple disabled elderly individuals simultaneously, leaving no extra time for them to “chew and savor” food. This situation leads to a lack of fulfilment in the food experience for disabled elderly individuals, *“I haven’t finished chewing this bite, and the next one is already being shoved into my mouth” (LK03).*

#### 3.3.3. Physical activity

From the perspective of elderly individuals with disabilities, engaging in physical exercise signifies their willingness to live life to the fullest. Regular exercise can reduce functional limitations, maximize independence, potentially slow the progression of diseases, and promote sleep. Many disabled elderly individuals exhibit a strong desire to improve their physical mobility. *“If you don’t move, your body deteriorates, and you end up like some of the people here, lying down, unable to sit up, it’s a vicious cycle” (LY10)*. Elderly care institutions invite professionals to provide exercise instruction tailored to the physical conditions of disabled elderly residents. Some disabled elderly individuals find this to be highly valuable, experiencing positive emotions and maintaining mental and physical well-being through exercise programmes or sports activities. *“They organize these activities for us. An expert comes to teach us to exercise. I participate as much as I can, and I feel better when I move” (LK08)*. However, most severely disabled elderly individuals exhibit disinterest and indifference towards this topic. This is because the staff, during their interactions while guiding and assisting with physical activities, fail to convey a sense of safety and confidence. As a result, the disabled elderly feel uncomfortable when engaging in these activities. *“They guide me through these exercises, many of which make me uncomfortable. They are too forceful and do not communicate with me at all” (LK13).*

### 3.4. Lonely soul

#### 3.4.1. Emotions

Elderly individuals in elderly care facilities often harbour a profound sense of uncertainty about the future. *“I have been eagerly anticipating the day when I can return home. Every time I ask them (my children), they never give me a straightforward answer” (LK02)*. While they yearn to return home, they also face inner struggles. Returning home would mean that their children would have to take on more caregiving responsibilities and make greater sacrifices. They do not want to burden their children further. *“I wish to die so that my children can go to work and live their lives without worry. I don’t want to suffer anymore” (LY07)*. Despite their desire for autonomy in controlling their lives, a paradox arises as they find themselves reliant on others’ assistance, often accompanied by condescension and discrimination. Most of the time, disabled elderly individuals find themselves alone, and the attentive presence of staff can alleviate their loneliness. *“I often sit alone recalling the past, reminiscing about my youth. I shared with a staff member about my younger days, how I loved singing and dancing. Once after dinner, that staff member invited me to sing a song for everyone. After I finished, everyone applauded, and it was truly fulfilling” (LY13)*. However, such opportunities to be heard are too rare. Elderly individuals with disabilities yearn to be needed, valued, and respected. They are often perceived as frail and nearing the end of life, rendering their existence seemingly meaningless in the eyes of others. The complexity of emotions is frequently overlooked and inadequately addressed, contributing to their profound suffering.

#### 3.4.2. Privacy

In most elderly care institutions, the majority of seniors reside in shared living spaces. The sole physical boundary distinguishing one living space from another is the curtain between beds. During daytime hours, most curtains remain open to facilitate continuous monitoring of the elderly by staff, yet this practice disrupts the privacy of disabled elderly individuals. *“When I wake up in the morning, I found the curtains drawn, which provoked considerable irritation in me, as it resulted in a complete lack of privacy” (LK10)*. Disabled elderly individuals perceive life in these care institutions as akin to residing in a public domain, where staff can freely enter their rooms and pass by their beds without restraint. Intimate activities such as bodily care and bathing, which are inherently private, are treated as public actions. *“He abruptly lifted my blanket to check if I had urinated, without regard for whether there were others around” (LY16)*. Disabled elderly individuals express a desire for these activities not to be made public, emphasizing, *“Don’t turn it into a public matter” (LK05)*. The neglect of privacy for elderly individuals with disabilities in care institutions leaves them feeling powerless to protect their own privacy. Living long-term in an environment that lacks privacy, they feel like “public objects.” However, due to their inability to independently manage personal care, they are forced to endure awkwardness and discomfort.

#### 3.4.3. Death and end-of-life experiences

Disabled elderly individuals in institutions face daily challenges of illness and pain, as well as passive activities such as idleness, sleeping, and waiting. They also suffer from a lack of valuable intimate relationships and companionship, ultimately awaiting death. *“The only thing I do is wait; I truly wish I were already dead” (LY13)*. In China, discussing death in front of terminally ill individuals is taboo, which leaves disabled elderly people to confront death alone. Some of these individuals claim they are fully prepared for death. *“I’m not afraid of dying. I’ve experienced everything in life, and I feel it was worth it” (LK04)*. However, most live in a state of uncertain fear. One individual remarked, *“I know my illness is incurable, and death is the next step. People tell me I will get better, but such well-meaning ‘deception’ is pointless. I don’t know how to cope. On one occasion, a volunteer attempted to discuss the topic of death with me. However, she was interrupted by the staff and prohibited from continuing the conversation(LY02)”*, Disabled elderly individuals are grappling with issues of life and death, such as what makes life valuable and how to face the end of life. The difficulty in finding answers to these questions causes significant distress, which persists every day until their death.

## 4. Discussion

This study elucidates the core concept of “maintaining symbiosis in conflict.” The research demonstrates that disabled elderly individuals in Chinese elderly care institutions encounter conflicts and contradictions in physical, psychological, and interpersonal dimensions. Nevertheless, they persistently sustain a balance between their own needs and the behaviours of others through patience and compromise, thereby achieving harmony within the elderly care institutions. Within this framework,our research has identified four fundamental dimensions, each comprising various sub-dimensions. These dimensions include the complex adaptation process, complexities in social interactions, physical pain, and the lonely soul. The varying evaluations of quality of life among different disabled elderly individuals highlight the complexity inherent in this subjective construct. Our delineation of these dimensions contributes to a more nuanced understanding of the factors influencing the quality of life perceptions in this demographic.

The fundamental dimension of the “Complex Adaptation Process” comprises four sub-dimensions: sense of identity, physical safety, self-esteem, and happiness. In China, elderly individuals experiencing functional decline often move from their homes to elderly care institutions. When the environment and services provided by these institutions surpass those at home, it can enhance their sense of identity (Fleming et al., 2016). Moreover, by comparing themselves with other residents, they construct new identities, find their place within the institutional environment, and integrate into the new setting more quickly.In line with Schenk et al.‘s concept of “meaningful activity”, elderly residents can derive a sense of purpose by participating in activities that not only make them happy but also bring joy to others (Hay et al., 2020; Schenk et al., 2013). Nevertheless, in our study, disabled elderly individuals may no longer be able to engage in meaningful activities for themselves. However, carefully planned meaningful activities can contribute to fostering a sense of “at-home” happiness (Shogren et al., 2017). The process of acclimating disabled elderly individuals to new environment may prompt shifts in their previously held values, as they develop new perspectives. This aligns with Mondaca et al.‘s findings that the preferences of elderly residents are not static but continually evolving (Mondaca et al., 2019). In our study, it is often observed that the staff and family members tend to overlook changes in the needs of disabled elderly individuals, particularly regarding their psychological and emotional needs. This negates the rights of disabled elderly individuals to alter their thoughts and make autonomous decisions, thereby constraining their current lives based on “past selves”. To a certain extent, it deprives them of the autonomy to control their lives. Consequently, when catering to the needs of disabled elderly individuals, it is imperative to strike a balance between “past selves” and “present selves.“ This necessitates the formulation of more individualized adaptation plans, (Heller et al., 2015), aimed at promoting a more proactive adaptation to the environment (O’Neill et al., 2022; Schepens et al., 2019).

The primary dimension of “Complex Social Interactions” comprises three sub-dimensions: interactions with the staff (Gautam et al., 2022) interactions with other elderly residents who are disabled, and interactions with family members. In this context, maintaining positive relationships with the staff is of paramount importance for enhancing the quality of life for disabled elderly residents (Roberts & Bowers, 2015). In the context of Chinese culture, the family members of disabled elderly individuals may employ the practice of gift-giving to garner more attention from their parents (Gautam et al., 2022). Previous research has emphasized the pivotal role of gift-giving in sustaining these connections, but it has not significantly fostered genuinely “intimate relationships” between disabled elderly residents and the staff (Schenk et al., 2013). Our study introduces a fresh perspective on understanding the relationships among disabled elderly residents, revealing their difficulties in forming interpersonal connections and even displaying instances of “bullying” behaviour among themselves, with the staff’s delayed interventions exacerbating their vulnerability (Gautam et al., 2022). In Chinese society, there exists a tradition of gift-giving, whereby staff members may unintentionally exhibit more concern and care towards elderly individuals and their families who offer gifts, while neglecting others. Elderly individuals and their families who do not offer gifts are more likely to receive less attention. This cultural backdrop exacerbates instances of “bullying” to some extent, further compromising the autonomy of certain elderly individuals with disabilities. When we consider the aspect of establishing relationships with family members, numerous scholars have affirmed the importance of emotional communication with family members in enhancing the quality of life (Haugan, 2022; Moreno-Fergusson et al., 2023). A decline in connections with family members results in feelings of abandonment, deception, and marginalization among elderly residents in elderly care institutions (Gautam et al., 2022). Our research further reveals that, despite facing various challenges in elderly care institutions, disabled elderly residents often maintain a façade of “rationality” and “restraint” to conceal their genuine feelings about the facility. They achieve this by preventing family members from visiting, thereby detaching themselves emotionally from their families.

In contrast to the challenge of establishing robust interpersonal networks in eldercare facilities, older individuals afflicted by multiple chronic illnesses, who grapple with the suffering imposed by medical conditions, prioritize the alleviation of physical pain and fatigue. Concurrently, they also express a keen desire for the satisfaction derived from food. Nutrition and dining have consistently been focal concerns for both the elderly residents and family members. Presently, the dietary options provided within eldercare facilities fall short of aligning with the preferences of these disabled elderly individuals (Hansen, 2020).

Despite contending with their illnesses, disabled elderly individuals aspire to promote physical activity to the best of their abilities and maintain a degree of independence, aligning with the findings of Schenk and colleagues (Schenk et al., 2013). However, within the bustling environment of eldercare facilities, staff members encounter significant constraints in facilitating self-directed choices for disabled elderly individuals. This limitation hampers their ability to meet the diverse needs of these individuals. Cater’s research unveils that elderly residents of eldercare facilities frequently describe their lives as “a sense of being institutionalized—akin to being in ‘jail,’ ‘prison,’ or ‘mice in our cages’” (Cater et al., 2022).

In addition to the progressive physical decline, disabled elderly individuals grapple with feelings of loneliness, having already forfeited their privacy within eldercare facilities, which exacerbates their negative emotions. This situation underscores the importance of staff members’ diligent management of issues such as incontinence and personal hygiene. Furthermore, they often find themselves in an environment where their voices and concerns appear to go unheard, exacerbating the sense of confronting death alone (Österlind et al., 2017). Disabled elderly individuals grapple with the desire for companionship from loved ones while trying to avoid becoming a burden to them. They concurrently seek meaning in life and endure the painful process of facing its end. This emotional conflict often results in feelings of helplessness, confusion, and distress. Studies indicate that the ability of disabled elderly individuals to articulate their thoughts and emotions about death is paramount in maintaining sense of existence as they approach the end of life (Dwyer et al., 2011). Consequently, Chinese eldercare must take into account the life trajectories of disabled elderly individuals, challenge the cultural taboos surrounding discussions of death, and underscore the pivotal role of end-of-life care for these individuals (Newberry et al., 2015). The struggle to find meaning in life and cope with the end of life makes it challenging for them to find answers

The study underscores the significant influence of societal values on the QoL of elderly individuals with disabilities residing in elderly care institutions. In the Chinese context, there is a prevailing collective ideology that places a lesser emphasis on individual autonomy and rights, particularly among disabled elderly individuals who prioritize collective interests over individual ones. Within Chinese culture, the value of harmonious social relationships is paramount, with “tolerance” serving as a means to attain such harmony. Consequently, disabled elderly residents in elderly care institutions are often inclined to adhere to directives. Despite gradually losing their autonomy in elderly care institutions, residents often strive to conceal their emotions and refrain from voicing dissent or taking actions that contradict their perception of what is right, in order to maintain the harmony and stability of the care institutions. Faced with instances of “bullying” behaviour from facility staff and other elderly residents, disabilities elderly individuals are often reluctant to express their feelings and are particularly averse to engaging in confrontations or conflicts. To avoid causing inconvenience to their children and caregivers, they frequently remain “silent” and bear their burdens alone. They tend to choose tolerance and compromise to avoid conflicts in social relationships and to maintain their “face” (Chou, 2010; Zhan et al., 2008). On the flip side, Chinese society places a high value on family, and many decisions, including those related to entering or leaving elderly care institutions, are made with the overarching goal of preserving family unity (Chou, 2010; Zhan et al., 2008). Fulfilling familial responsibilities holds heightened significance within the collectivist framework of Chinese society (Fuligni et al., 2002). These decisions are driven by the desire to minimize the guilt and shame that children might experience and to make a final contribution to the family by sacrificing their well-being. Likewise, the aspirations of disabled elderly individuals for a gratifying later life are deeply rooted in the concept of the “family,” thus ensuring the harmonious and stable functioning of elderly care institutions.

The grounded theory methodology necessitates a thorough literature review following data analysis (Corbin & Strauss, 1990). This study presents findings not previously discussed in existing research, demonstrating that the unique Chinese concepts of “family” and “collectivism” are crucial foundations for the theme of “Maintaining symbiosis in conflict.” These concepts facilitate the adaptation and acceptance of elderly care institutions by disabled elderly individuals and contribute to the harmonious environment within these institutions. Further research is needed to verify the potential relationships between these unique Chinese concepts and other dimensions identified in this study.

## 5. Limitations

The present study has several limitations. Firstly, the study focused on disabled elderly individuals in urban care institutions. The findings of this study may not apply to disabled elderly individuals in rural care institutions. Therefore, future attention should be given to the quality of life and improvement strategies for disabled elderly individuals in rural care institutions. Secondly, the interrelationships and significance among various dimensions cannot be precisely measured. Therefore, the research conclusions in this study necessitate further validation through quantitative research methods.

## 6. Conclusion

In this study, we adopt a perspective centred on disabled elderly individuals, presenting multiple dimensions of the QoL for disabled elderly residents in elderly care institutions. Research indicates that even for the most vulnerable disabled elderly individuals, the responsibility of enhancing their quality of life extends beyond the purview of personnel within elderly care institutions; instead, it is a collective responsibility shared by all members of society. In practical terms, government entities should moderately increase financial support for and quality oversight of elderly care institutions. Administrative personnel within these institutions should optimize regulatory frameworks, it is crucial to establish a mechanism for caregiver feedback and complaints, which is linked to staff performance evaluations. With a particular emphasis on addressing and resolving instances of “bullying” within these settings. The government should actively strengthen death education throughout society via public outreach and media campaigns, gradually eradicating cultural taboos surrounding death. Additionally, elderly care institutions should prioritize the needs of disabled elderly individuals by developing personalized service plans and regularly assessing changes to dynamically adjust service provision. Concurrently, these institutions should appropriately augment staffing levels and foster interaction between disabled elderly individuals and staff through meaningful activities to facilitate the establishment of amicable relationships. Additionally, elderly care institutions should enhance staff training in end-of-life education, actively engage with professional social organizations to jointly provide end-of-life care support services, and proactively address the emotional needs of disabled elderly individuals when facing death. Family members of disabled elderly residents should increase the frequency of in-person visits, providing additional emotional support for disabled elderly individuals.

## References

[cit0001] Bowling, A., Gabriel, Z., Dykes, J., Dowding, L. M., Evans, O., Fleissig, A., Banister, D., & Sutton, S. (2003). Let’s ask them: A national survey of definitions of quality of life and its enhancement among people aged 65 and over. *International Journal of Aging & Human Development*, 56(4), 269–12. 10.2190/BF8G-5J8L-YTRF-6404 14738211

[cit0002] Bradley, D. E. (2011). Litwak and Longino’s developmental model of later-life migration: Evidence from the American community survey, 2005–2007. *Journal of Applied Gerontology*, 30(2), 141–158. 10.1177/0733464810386463

[cit0003] Brandburg, G. L., Symes, L., Mastel‐Smith, B., Hersch, G., & Walsh, T. (2013). Resident strategies for making a life in a nursing home: A qualitative study. *Journal of Advanced Nursing*, 69(4), 862–874. 10.1111/j.1365-2648.2012.06075.x 22812933

[cit0004] Burke, W. J. (1996). The clinical care of the aged person: An interdisciplinary perspective - Satin,DG. *The American Journal of Geriatric Psychiatry*, 4(2), 183–184. 10.1097/00019442-199621420-00013

[cit0005] Cater, D., Tunalilar, O., White, D. L., Hasworth, S., & Winfree, J. (2022). “Home is home:” Exploring the meaning of home across long-term care settings. *Journal of Aging and Environment*, 36(3), 321–338. 10.1080/26892618.2021.1932012

[cit0006] China Civil Affairs Statistical Yearbook. (2021). Retrieved August 5, 2021, from https://www.mca.gov.cn/images3/www2017/file/202208/2021mzsyfztjgb.pdf

[cit0007] Chou, R.-A. (2010). Willingness to live in eldercare institutions among older adults in urban and rural China: A nationwide study. *Ageing and Society*, 30(4), 583–608. 10.1017/S0144686X09990596

[cit0008] Corbin, J., & Strauss, A. (1990). Grounded theory research - procedures, canons and evaluative criteria. *Zeitschrift Fur Soziologie*, 19(6), 418–427. 10.1515/zfsoz-1990-0602

[cit0009] Dwyer, L. L., Hansebo, G., Andershed, B., & Ternestedt, B. M. (2011). Nursing home residents’ views on dying and death: Nursing home employee’s perspective. *International Journal of Older People Nursing*, 6(4), 251–260. 10.1111/j.1748-3743.2010.00237.x 21631874

[cit0010] Engward, H. (2013). Understanding grounded theory. *Nursing Standard (Through 2013)*, 28(7), 37. 10.7748/ns2013.10.28.7.37.e7806 24128248

[cit0011] Fleming, R., Goodenough, B., Low, L.-F., Chenoweth, L., & Brodaty, H. (2016). The relationship between the quality of the built environment and the quality of life of people with dementia in residential care. *Dementia (London, England)*, 15(4), 663–680. 10.1177/1471301214532460 24803645

[cit0012] Francis, J. J., Johnston, M., Robertson, C., Glidewell, L., Entwistle, V., Eccles, M. P., & Grimshaw, J. M. (2010). What is an adequate sample size? Operationalising data saturation for theory-based interview studies. *Psychology & Health*, 25(10), 1229–1245. 10.1080/08870440903194015 20204937

[cit0013] Fuligni, A. J., Yip, T., & Tseng, V. (2002). The impact of family obligation on the daily activities and psychological well‐being of Chinese American adolescents. *Child Development*, 73(1), 302–314. 10.1111/1467-8624.00407 14717259

[cit0014] Gautam, S., Montayre, J., & Neville, S. (2022). Seeking and maintaining connections: A grounded theory study of maintaining spirituality in residential aged care facilities. *International Journal of Older People Nursing*, 17(3), e12435. 10.1111/opn.12435 34793613

[cit0015] Glaser, B. G., & Strauss, A. L. (1965). Discovery of substantive theory: A basic strategy underlying qualitative research. *The American Behavioral Scientist*, 8(6), 5–12. 10.1177/000276426500800602

[cit0016] Hallberg, L. R. (2006). The “core category” of grounded theory: Making constant comparisons. *International Journal of Qualitative Studies on Health and Well-Being*, 1(3), 141–148. 10.1080/17482620600858399

[cit0017] Hansen, K. V. (2020). Older adults—Their focus on food and future living: A grounded theory approach. *The Qualitative Report*. 10.46743/2160-3715/2020.3678

[cit0018] Haugan, G. (2022). Spiritual and existential care in nursing homes. *Obzornik Zdravstvene Nege*, 56(4), 240–245. 10.14528/snr.2022.56.4.3196

[cit0019] Hay, M. E., Mason, M. E., Connelly, D. M., Maly, M. R., & Laliberte Rudman, D. (2020). Pathways of participation by older adults living in continuing care homes: A constructivist grounded theory study. *Activities, Adaptation & Aging*, 44(1), 1–23. 10.1080/01924788.2019.1581021

[cit0020] He, W., Goodkind, D., & Kowal, P. R. (2016). *An aging world: 2015*. United States Census Bureau.

[cit0021] Heller, T., Gibbons, H. M., & Fisher, D. (2015). Caregiving and family support interventions: Crossing networks of aging and developmental disabilities. *Intellectual & Developmental Disabilities*, 53(5), 329–345. 10.1352/1934-9556-53.5.329 26458169

[cit0022] Johnson, C. W., & Parry, D. C. (2022). *Fostering social justice through qualitative inquiry: A methodological guide*. Taylor & Francis.

[cit0023] Kane, R. L., Rockwood, T., Hyer, K., Desjardins, K., Brassard, A., Gessert, C., & Kane, R. (2005). Rating the importance of nursing home residents’ quality of life. *Journal of the American Geriatrics Society*, 53(12), 2076–2082. 10.1111/j.1532-5415.2005.00493.x 16398890

[cit0024] Lucas-Carrasco, R., Laidlaw, K., & Power, M. J. (2011). Suitability of the WHOQOL-BREF and WHOQOL-OLD for Spanish older adults. *Aging & Mental Health*, 15(5), 595–604. 10.1080/13607863.2010.548054 21815852

[cit0025] Mondaca, M., Josephsson, S., Borell, L., Katz, A., & Rosenberg, L. (2019). Altering the boundaries of everyday life in a nursing home context. *Scandinavian Journal of Occupational Therapy*, 26(6), 441–451. 10.1080/11038128.2018.1483426 29938554

[cit0026] Moreno-Fergusson, M. E., Caez-Ramírez, G. R., Sotelo-Díaz, L. I., & Sánchez-Herrera, B. (2023). Nutritional care for institutionalized persons with dementia: An integrative review. *International Journal of Environmental Research & Public Health*, 20(18), 6763. 10.3390/ijerph20186763 37754622 PMC10531301

[cit0027] National Bureau of Statistics. (2021, May 11). The 7th national population census bulletin. https://www.stats.gov.cn/zt_18555/zdtjgz/zgrkpc/dqcrkpc/

[cit0028] Newberry, G., Martin, C., & Robbins, L. (2015). How do people with learning disabilities experience and make sense of the ageing process? *British Journal of Learning Disabilities*, 43(4), 285–292. 10.1111/bld.12149

[cit0029] O’Neill, M., Ryan, A., Tracey, A., & Laird, L. (2022). ‘Waiting and wanting’: Older peoples’ initial experiences of adapting to life in a care home: A grounded theory study. *Ageing and Society*, 42(2), 351–375. 10.1017/S0144686X20000872

[cit0030] O’neill, T., Felsenberg, D., Varlow, J., Cooper, C., Kanis, J., & Silman, A. (2020). The prevalence of vertebral deformity in European men and women: The European vertebral osteoporosis study. *Journal of Bone and Mineral Research*, 11(7), 1010–1018. 10.1002/jbmr.5650110719 8797123

[cit0031] Österlind, J., Ternestedt, B. M., Hansebo, G., & Hellström, I. (2017). Feeling lonely in an unfamiliar place: Older people’s experiences of life close to death in a nursing home. *International Journal of Older People Nursing*, 12(1), e12129. 10.1111/opn.12129 27624362

[cit0032] Outline of the Fourteenth Five-Year Plan for the National Economic and Social Development of the People’s Republic of China and the Vision 2035. Retrieved March 13, 2021, from https://www.gov.cn/xinwen/2021-03/13/content_5592681.htm

[cit0033] Pequeno, N. P. F., NldA, C., Marchioni, D. M., Lima, S. C. V. C., & Lyra Cd, O. (2020). Quality of life assessment instruments for adults: A systematic review of population-based studies. *Health and Quality of Life Outcomes*, 18(1), 1–13. 10.1186/s12955-020-01347-7 32605649 PMC7329518

[cit0034] Rijnaard, M., Van Hoof, J., Janssen, B., Verbeek, H., Pocornie, W., Eijkelenboom, A., Beerens, H., Molony, S., & Wouters, E. (2016). The factors influencing the sense of home in nursing homes: A systematic review from the perspective of residents. *Journal of Aging Research*, 2016, 1–16. 10.1155/2016/6143645 PMC4893593 27313892

[cit0035] Roberts, T., & Bowers, B. (2015). How nursing home residents develop relationships with peers and staff: A grounded theory study. *International Journal of Nursing Studies*, 52(1), 57–67. 10.1016/j.ijnurstu.2014.07.008 25443304 PMC4258422

[cit0036] Sandgren, A., Arnoldsson, L., Lagerholm, A., & Bökberg, C. (2021). Quality of life among frail older persons (65+ years) in nursing homes: A cross‐sectional study. *Nursing Open*, 8(3), 1232–1242. 10.1002/nop2.739 34482652 PMC8046081

[cit0037] Schenk, L., Meyer, R., Behr, A., Kuhlmey, A., & Holzhausen, M. (2013). Quality of life in nursing homes: Results of a qualitative resident survey. *Quality of Life Research*, 22(10), 2929–2938. 10.1007/s11136-013-0400-2 23595411

[cit0038] Schepens, H. R., Van Puyenbroeck, J., & Maes, B. (2019). How to improve the quality of life of elderly people with intellectual disability: A systematic literature review of support strategies. *Journal of Applied Research in Intellectual Disabilities*, 32(3), 483–521. 10.1111/jar.12559 30575226

[cit0039] Shogren, K. A., Wehmeyer, M. L., Lassmann, H., & Forber-Pratt, A. J. (2017). Supported decision making: A synthesis of the literature across intellectual disability, mental health, and aging. *Education and Training in Autism and Developmental Disabilities*, 52(2), 144–157.

[cit0040] Sima, R.-M., Pleş, L., Socea, B., Sklavounos, P., Negoi, I., Stănescu, A.-D., Iordache, I.-I., Hamoud, B. H., Radosa, M. P., Juhasz‑Boess, I., Solomayer, E., Dimitriu, M., Cîrstoveanu, C., Şerban, D., & Radosa, J. (2021). Evaluation of the SF‑36 questionnaire for assessment of the quality of life of endometriosis patients undergoing treatment: A systematic review and meta‑analysis. *Experimental and Therapeutic Medicine*, 22(5), 1–14. 10.3892/etm.2021.10718 PMC8461506 34630638

[cit0041] Sion, K. Y., Verbeek, H., Zwakhalen, S. M., Odekerken-Schröder, G., Schols, J. M., & Hamers, J. P. (2020). Themes related to experienced quality of care in nursing homes from the resident’s perspective: A systematic literature review and thematic synthesis. *Gerontology and Geriatric Medicine*, 6, 2333721420931964. 10.1177/2333721420931964 32637461 PMC7318818

[cit0042] Strauss, A., & Corbin, J. (1998). Basics of qualitative research techniques.

[cit0043] Suddaby, R. (2006). *From the editors: What grounded theory is not* (Vol. 49, pp. 633–642). Academy of Management Briarcliff Manor.

[cit0044] Toh, H. J., Yap, P., Wee, S. L., Koh, G., & Luo, N. (2021). Feasibility and validity of EQ-5D-5L proxy by nurses in measuring health-related quality of life of nursing home residents. *Quality of Life Research*, 30(3), 713–720. 10.1007/s11136-020-02673-5 33067756

[cit0045] Vanleerberghe, P., De Witte, N., Claes, C., Schalock, R. L., & Verté, D. (2017). The quality of life of older people aging in place: A literature review. *Quality of Life Research*, 26(11), 2899–2907. 10.1007/s11136-017-1651-0 28707047

[cit0046] Wuetherick, B. (2010). Basics of qualitative research: Techniques and procedures for developing grounded theory. *Canadian Journal of University Continuing Education*, 36(2). 10.21225/D5G01T

[cit0047] Yang, Y., & Meng, Y. (2020). Is China moving toward healthy aging? A tracking study based on 5 phases of CLHLS data. *International Journal of Environmental Research & Public Health*, 17(12), 4343. 10.3390/ijerph17124343 32560573 PMC7345691

[cit0048] Zhan, H. J., Feng, X., & Luo, B. (2008). Placing elderly parents in institutions in urban China: A reinterpretation of filial piety. *Research on Aging*, 30(5), 543–571. 10.1177/0164027508319471

